# Studies on the active components and antioxidant activities of the extracts of *Mimosa pudica* Linn. from southern China

**DOI:** 10.4103/0973-1296.75899

**Published:** 2011

**Authors:** Jing Zhang, Ke Yuan, Wen-long Zhou, Jian Zhou, Ping Yang

**Affiliations:** 1 *The Nurturing Station for the State Key Laboratory of Subtropical Silviculture, Zhejiang Agriculture and Forestry University, Lin’an-311 300, P.R*; 2 *College of Pharmacy, Henan University of Traditional Chinese Medicine, Zhengzhou-450 008, P.R.China*

**Keywords:** Antioxidant activity, DPPH, ferric reducing/antioxidant power, *Mimosa pudica* Linn., total flavonoid, total phenolic

## Abstract

**Background:**

The total flavonoid (TF) and total phenolic (TP) contents of the ethanol extracts of the whole plant, stem, leaf, and seed of *Mimosa pudica* Linn belonging to the genus *Mimosa* (Family: Fabaceae alt. Leguminosae), which originates from the subtropical regions of southern China, were determined in this experiment.

**Materials and Methods:**

The antioxidant activity of the extracts and 5 flavonoid monomers of *M. pudica* Linn. were also evaluated by 2 assays, the 1,1-diphenyl-2-picrylhydrazyl (DPPH) radical-scavenging activity and ferric reducing/antioxidant power (FRAP) assays. In addition, correlation analysis was also made in the present study.

**Results:**

The results showed that leaf extracts contained the highest amount of TF and TP, and the content was significantly higher than that found in other parts of the plant. Moreover, the sequence of antioxidant activity of the ethanol extracts was as follows: leaf > the whole plant > seed > stem; the sequence of the 5 flavonoid monomers was as follows: 5,7,3´,4´-tetrahydroxy-6-C-[β-D-apiose-(1→4)]-β-D-glycopyranosyl flavone **(1)** > isorientin **(2)** > orientin **(3)** > isovitexin **(4)** > vitexin **(5)**, and the antioxidant activity of compound 1 is equivalent to the synthetic antioxidant trolox or a bit stronger than trolox, and significant correlations were found among the active ingredient contents and the results of antioxidant activity.

**Conclusion:**

The present study suggested that *M. pudica* Linn. could be a potential rich source of natural antioxidants.

## INTRODUCTION

The aging of human organs, cancer, immune dysfunction, and other diseases are closely related to the oxidative damage in cells induced by free radicals, and antioxidants play an important role in inhibiting and scavenging free radicals, thus it can provide protection for human beings. Synthetic antioxidants are commercially available but quite unsafe, and their toxicity is a worrying problem. The natural antioxidants, especially phenolics and flavonoids, are safe and they possess biological activity, so the current researcher are now directed toward natural antioxidants from natural plants. Flavonoids and phenolics play an important role in the prevention of cardiovascular disease, aging, and effectively scavenging oxygen free radicals, and most of them possess antioxidant activity.[1–4]

*Mimosa pudica* Linn. belongs to the genus *Mimosa* (Family: Fabaceae alt. Leguminosae) is popularly named as a sensitive and shy plant, and the whole plant of *M. pudica* Linn. has been used as a traditional medicine for the treatment of neurasthenia, insomnia, traumatic injury, pulmonary tuberculosis, and others. *M. pudica* Linn. mainly grows on the hillside, jungle, glade, and roadside of Asia. Many previous studies on *M. pudica* Linn. revealed the presence of flavonoids, phenolics, and others.[5–9] In addition, many bioactivities of *M. pudica* Linn. were also studied, such as antioxidant, antibacterial, antihepatotoxic activities, and so on.[10–13] The objective of the present study was to evaluate the antioxidant activity *in vitro*. Considering the various mechanisms of antioxidant action, 1,1-diphenyl-2-picrylhydrazyl (DPPH) and ferric reducing/antioxidant power (FRAP) assays were both used to evaluate the antioxidant activities of *M. pudica* Linn. extracts in this study, so as to more accurately reflect antioxidant action.

## MATERIALS AND METHODS

### Instruments and reagent

Infinite M 200 Universal Microplate Spectrophotometer (Swiss Tecan Company, Switzerland) was used to measure the absorbance (DPPH and FARP assays) and UV-2102 PCS UV–Vis spectrophotometer (Shanghai Unica Company Ltd, China) was used to measure the absorbance of NaNO_2_–Al(NO3)_3_ chromogenic method and Folin–Ciocalteu method. All reagents were of analytical grade. Fe3^+^–tripyradyltriazine (TPTZ), DPPH, 6-hydroxy-2,5,7,8-tetramethychroman-2-carboxylic acid (Trolox) were all purchased from Sigma (USA). Standards of rutin and gallic acid were purchased from China Pharmaceutical and Biological Products Testing Station. (The batch numbers were 10080-200306 and 110831-200302, respectively.) Compounds **1, 2, 3, 4, 5** were all isolated in our laboratory, and their structures were determined by ^1^H-NMR, ^13^C-NMR and MS, and the purities were calibrated by normalization method (purities > 98%).

### Plant materials and extraction

*M. pudica* Linn. was collected in Sanya City, Hainan Province, China, and was identified as the whole plant of *M. pudica* Linn. of *Mimosa* genus belonging to the family Leguminosae by Shi-man Huang, Professor of Plant taxonomy of the Hainan University, and the specimen were deposited in our laboratory. The dried plants were powdered and passed through sieve no. 40. The dried powder of the whole plants, leaves, seeds, and stems of *M. pudica* Linn. (1.0 g) were weighed, and were extracted in Ultrasonic cleaner at 25°C, with 15 times of 95% ethanol for 3 times (40 min each time), then the extracts were concentrated to dryness by the rotary evaporator, and then dissolved them to 10-mL volumetric flasks, using 70% ethanol. Compounds **1, 2, 3, 4, 5** (1.5 mg) were weighed and dissolved to 5-mL volumetric flasks, using 70% ethanol, respectively. Finally, put the sample solutions into a 4°C refrigerator until use.

### Quantification of TF and TP contents

The TF content was determined according to the NaNO_2_–Al(NO_3_)_3_ method.[14] The standard curve was obtained using rutin concentration (*X*) as the abscissa axis and absorption values (*Y*) as the vertical axis, *Y* = 130.49*X* − 0.0139, *R*^2^ = 0.9991. The results were expressed as rutin equivalent (RE).

The TP content was determined according to the Folin–Ciocalteu method.[10] The standard curve was obtained using gallic acid concentration (*X*) as the abscissa axis and absorption values (*Y*) as the vertical axis, *Y* = 94.4578*X* + 0.1293, *R*^2^ = 0.998. The results were expressed as gallic acid equivalent (GAE).

### Determination of antioxidant activity

#### DPPH radical-scavenging activity

The DPPH radical-scavenging activity was evaluated according to the literature[15] with a slight modification. Briefly, 100 μL of each sample (with various concentrations) was added to 200 μL of DPPH ethanol solution. After mixing gently and standing at 24°C for 30 min, the absorbance was measured at 530 nm using a VERSAmax(Tecan Group Ltd., Männedorf, Switzerland) microplate reader spectrophotometer. The percentage of DPPH radical-scavenging was calculated using the following formula: DPPH radical-scavenging rate (%) = 100 (1 − (*A*_p_ − *A*_c_)/*A*_max_) (with *A*: absorbance). Here, *A*_p_ was the stable absorbance of DPPH ethanol solution (200 μL) plus sample solution (100 μL), Ac was the stable absorbance of 70% ethanol solution (200 μL) plus sample solution (100 μL) and *A*_max_ was the stable absorbance of DPPH ethanol solution (200 μL) plus 70% ethanol solution (100 μL).

### Ferric reducing/antioxidant power

The reducing power of *M. pudica* Linn. was measured using FRAP assay.[16] The reaction was carried out in a microtiter plate. Each sample (10 μL) was added to 300 μL of FRAP reagent (10 parts of 0.3 mol/L sodium acetate buffer at pH 3.7, 1 part of 0.01 mol/L TPTZ solution, and 1 part of 0.02 mol/L FeCl_3_· 6H_2_O solution), and 70% ethanol (10 μL) plus FRAP reagent (300 μL) was used as a blank, then the absorbance at 593 nm was read after 10 min. Fresh working solutions of trolox were used for calibration, and the standard curve was obtained using Trolox concentration (0.06–0.30 mg/mL) as the abscissa axis and absorption values as the vertical axis, *Y* = 3.7939*X* − 0.0435, (*r*^2^ = 0.997). The total antioxidant capacity was calculated from the standard curve and expressed as Trolox equivalent antioxidant capacity (TEAC) values, briefly, TE (mg/g).

### Statistical analysis

All the data were presented as mean ± standard deviations of 3 determinations. Pearson’s correlation test was used to assess correlations between data by using the SPSS system version 13.0(SPSS Inc., Chicago, USA) for Windows, and the figures were produced by using OriginPro7.5 (OriginLab Corporation, Northampton, USA.).

## RESULTS AND DISCUSSION

### TF and TP contents

*M. pudica* Linn. has more flavonoids and phenolics, and they are secondary metabolites widespread in plants, and have a variety of biological activities, especially an important role in the prevention of cardiovascular disease, aging, and effectively scavenging oxygen free radicals. The contents of the TF and TP of the extracts were determined according to NaNO_2_–Al(NO_3_) and Folin-Ciocalteu methods, and were expressed as RE and GAE, the data are shown in Table 1. Results from this table show that the leaf extract contained the highest amount of TF and TP, and they were significantly higher than other parts, whereas there were little differences among TF and TP contents in the other parts, and the contents of TF were significantly higher than TP in all the extracts.

**Table 1:** The contents of total flavonoids and total phenolics in the *Mimosa pudica* Linn. extracts

Samples	TF (mg/g)	TP (mg/g)
The whole plant	0.42 ± 0.03	2.55 ± 0. 33
Stem	0.34 ± 0.02	2.58 ± 0. 51
Leaf	0.81 ± 0.02	9.87 ± 0.07
Seed	0.43 ± 0.03	2.31 ± 0.04

TP: Total phenolics, TF: Total flavonoids, Values expressed as means ± standard deviation (n = 3)

### DPPH radical-scavenging activity

Free radicals are required for normal cell function at physiologic concentration, but excessive amount of free radicals can damage cellular components, such as lipids, protein, and DNA.[17] Thus, the free radical-scavenging activities of antioxidants can protect the human body from serious damage by free radicals and retard the progress of many chronic diseases.

**Figure 1:**
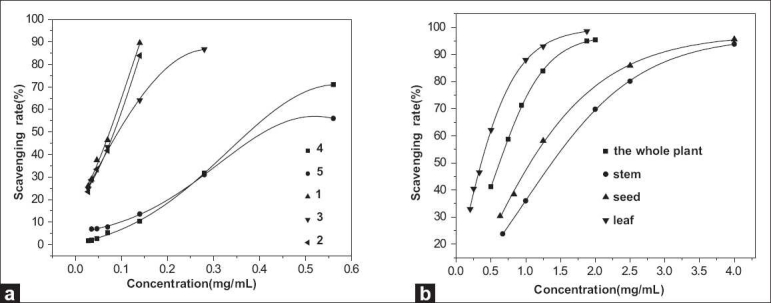
The scavenging rate of the 5 flavonoid monomers and the extracts on DPPH● with different concentrations. (a: the 5 flavonoid monomers; b: the extracts)

The DPPH assay has been widely used to test the free radical-scavenging ability of plants. In the present study, DPPH assay was used for determining the scavenging activity on free radicals. The DPPH scavenging activity of the 5 flavonoid monomers and *M. pudica* Linn. extracts are shown in Figure 1. The data showed that excessive concentration would clear the free radicals thoroughly, and too low a concentration could not detect the scavenging activity very well, but the scavenging activity increased with the increase in sample concentration in their proper concentration scope. Therefore, to compare the antioxidant capacity of these substances better, the half maximal inhibitory concentration (IC_50_) value is chosen as a parameter, and IC_50_ value is the concentration of the extracts when the scavenging rate is 50%. The IC_50_ values were shown in Table 2, and they showed that the sequence of IC_50_ values of the ethanol extracts was as follows: leaf > the whole plant > seed > stem; the sequence of IC_50_ values of the 5 flavonoid monomers was as follows: **1 > 2 > 3 > 4 > 5**.

**Table 2:** The determine results of IC_50_ and TE values of flavonoid monomers and the extracts with different concentrations

	Sample									
	The whole plant	Stem	Leaf	Seed	4	5	2	3	1	Trolox
IC50 (mg/ mL)	0.61 ± 0.03	1.39 ± 0.05	0.35 ± 0.01	1.07 ± 0.02	0.37 ± 0.01	0.41 ± 0.04	0.08 ± 0.01	0.09 ± 0.01	0.07 ± 0.01	0.07 ± 0.01
TE (mg/g)	11.64 ± 0.12	3.20 ± 0.09	22.36 ± 0.19	3.55 ± 0.01	129.12 ± 0.27	116.68 ± 0.55	947.15 ± 0.46	783.11 ± 0.71	1200 ± 0.22	1000 ± 0.67

IC_50_, the half maximal inhibitory concentration; TE: Trolox equivalent, Values expressed as means ± standard deviation (n = 3)

### Total antioxidant activity

**Figure 2:**
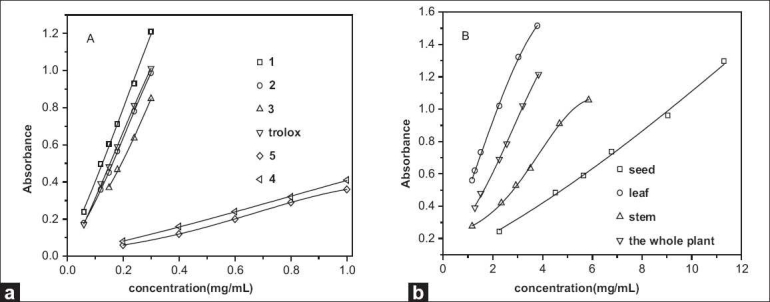
The absorbance of the 5 flavonoid monomers and the extracts with different concentrations. (a: the 5 flavonoid monomers; b: the extracts)

FRAP assay is based on the ability to reduce Fe^3+^ to Fe^2+^ in the presence of TPTZ, and with the formation of more stable intense blue Fe^2+^–TPTZ with an absorption maximum at 593 nm, autoxidation chain reactions were interrupted, so causing an increase of absorbance. Many studies show that the reducing power of substances is closely related to antioxidant activity, the greater the reducing power, the stronger the antioxidant activity;[18] therefore, the reducing power can reflect the antioxidant activity. FRAP assay can be used for detecting the total antioxidant activity. The absorbance of the 5 flavonoid monomers and the extracts from *M. pudica* Linn. are shown in Figure 2; it can be found that the absorbance increased with the increasing of sample concentration in experimental scope. In this article, the results are expressed as TE values, which are shown in Table 2. The sequence of reducing power of the ethanol extracts was as follows: leaf > the whole plant > seed > stem, and TE value of leaf is (22.367 ± 0.512) mg/g, and it means that the antioxidant activity of 1 g dried sample is equivalent to (22.367 ± 0.512) mg Trolox. The sequence of reducing power of the 5 flavonoid monomers was as follows: **1> 2> 3 > 4 > 5**, TE value of 1 is (1.200 ± 0.014) mg/mg, and it means that the antioxidant activity of 1 mg compound **1** is equivalent to (1.200 ± 0.014) mg Trolox.

### Relativzity analysis between the TF and TP contents and antioxidant capacity of the extracts

In the present study, the relativity analysis among the TF and TP contents and antioxidant activity of the extracts is made, and the data are shown in Table 3. In this result, the significant negative correlation between IC_50_ and TEAC values indicated that the extracts were capable of scavenging free radicals and reducing Fe^3+^, and both the 2 assays could be used for the measurement of antioxidant activity of *M. pudica* Linn. *in vitro*. The significant correlation between the antioxidant activity of extracts and the active ingredient contents indicated that flavonoids and phenolics played an important role in antioxidant activity of *M. pudica* Linn.

**Table 3:** Relativity analysis between the total flavonoids and total phenolics contents and antioxidant capacity of the extracts

	TP content	TF content	DPPH (IC_50_)	FRAP (TE)
TP content	1	0.977**	–0.807*	0.921**
TF content		1	–0.722*	0.906**
DPPH (IC_50_)			1	–0.929**
FRAP (TE)				1

TP: Total phenolics, TF: Total flavonoids, DPPH: 1,1-diphenyl-2-picrylhydrazyl, FRAP: Ferric reducing/antioxidant power, IC_50_: The half maximal inhibitory concentration, TE: Trolox equivalent

* Correlation is significant at the 0.05 level (2-tailed)

** Correlation is significant at the 0.01 level (2-tailed)

### Relationship of the structure of flavonoid monomers and the antioxidant activity

Flavonoids with phenolic hydroxyl groups is able to provide electronic (hydrogen) to free radical lipid peroxidation (LOO·), which transforms it into a more stable lipid peroxide (LOOH), thus interrupting the radical chain reaction; therefore, phenolic hydroxyls in the structure of flavonoids are the key factors of antioxidant activity.[19] The literature[20] reported that B ring of flavonoid was the crucial site and 4´-OH was the main base, and they almost determined the strength of antioxidant activity. 3´-OH, 5´-OH, 7-OH, and 5-OH were synergistic bases when 4´-OH was present. In the present study, the 5 flavonoid monomers all have 7-OH, 5-OH, and 4´-OH, and **2** and **4** have 3´-OH compared with **3** and **5**, so the antioxidant activity of the former one is stronger than the latter. It is consistent with the reported literature. In addition, **1** is a two-glycosides, and its antioxidant activity was stronger than the others. This phenomenon suggested that antioxidant activity may be increased slightly with the growth of the carbon chain of sugar in flavonoids. Furthermore, according to the small differences between **4** and **3** (**4** and **5**), we could find that the location of sugar in the A ring had little influence on their antioxidant activity of flavonoids.

## CONCLUSION

As the research of the plant antioxidant activity continue in-depth, more and more natural antioxidant components, which relate to cosmetics, health products, food, and medicine, show that the natural antioxidants have increasingly broad prospects of development. The present study found that the whole plant, stems, leaves, and seeds of *M. pudica* Linn. showed strong antioxidant capacity, and leaf extract is the strongest, and stem extracts are the weakest. Moreover, we might speculate that the antioxidant activity of *M. pudica* Linn. *in vitro* could be related to the high concentration of flavonoids and phenolics, and the antioxidant activity of the 3 flavonoid monomers (**1, 2, 3**) are similar to the positive control (trolox). So it can be known that *M. pudica* Linn. may provide potential natural antioxidants for the medicine industry and other fields.
